# Supplementary figures and images for: Antitumor activity of Chlorella sorokiniana and Scenedesmus sp. microalgae native of Nuevo León State, México
Source: PeerJ. 2018 Feb 9;6:e4358. doi: 10.7717/peerj.4358 (PMC5808310; doi:10.7717/peerj.4358)

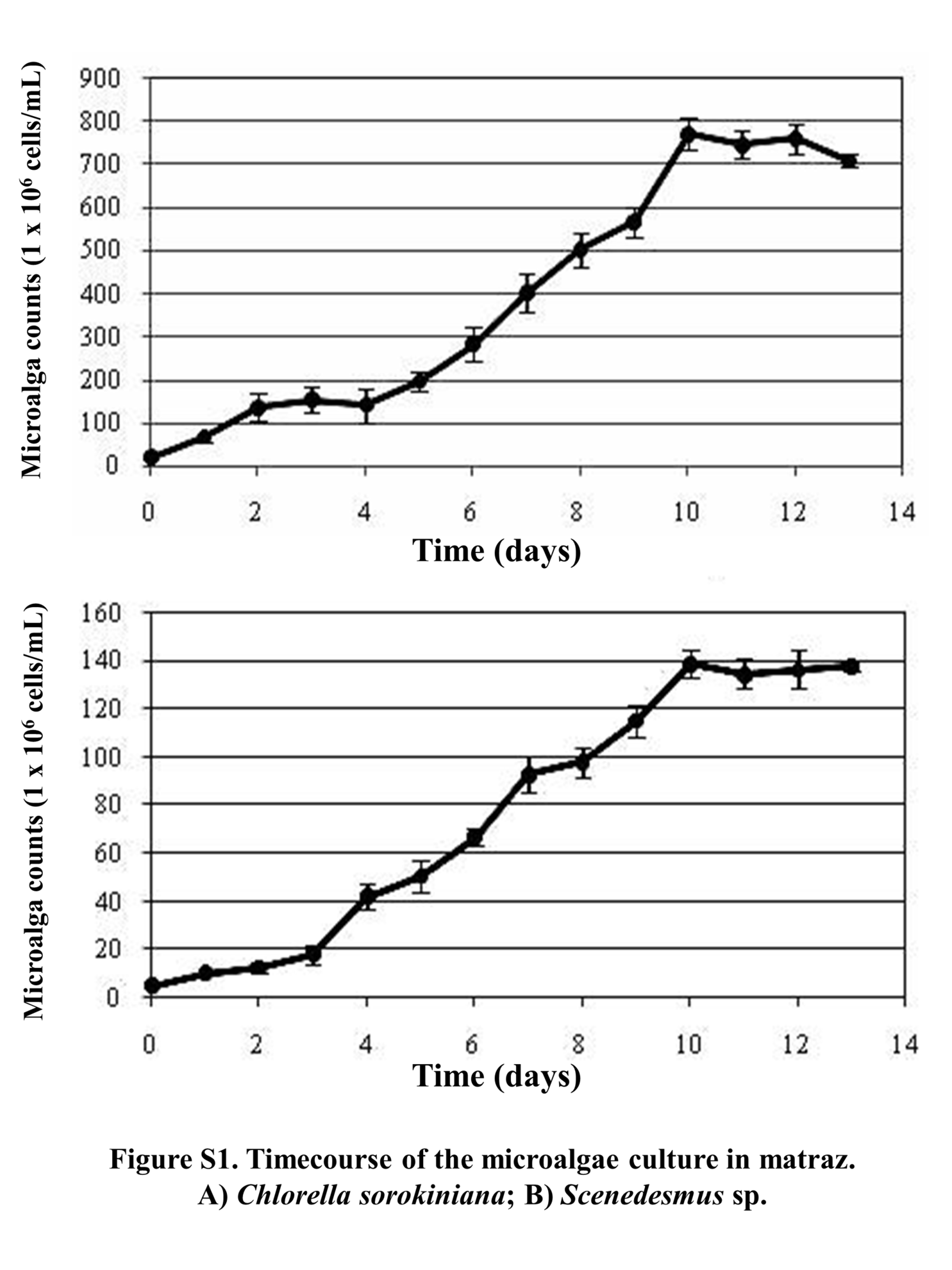

Supplement: Figure S1 — (A) Chlorella sorokiniana; (B) Scenedesmus sp. [file peerj-06-4358-s001.png]

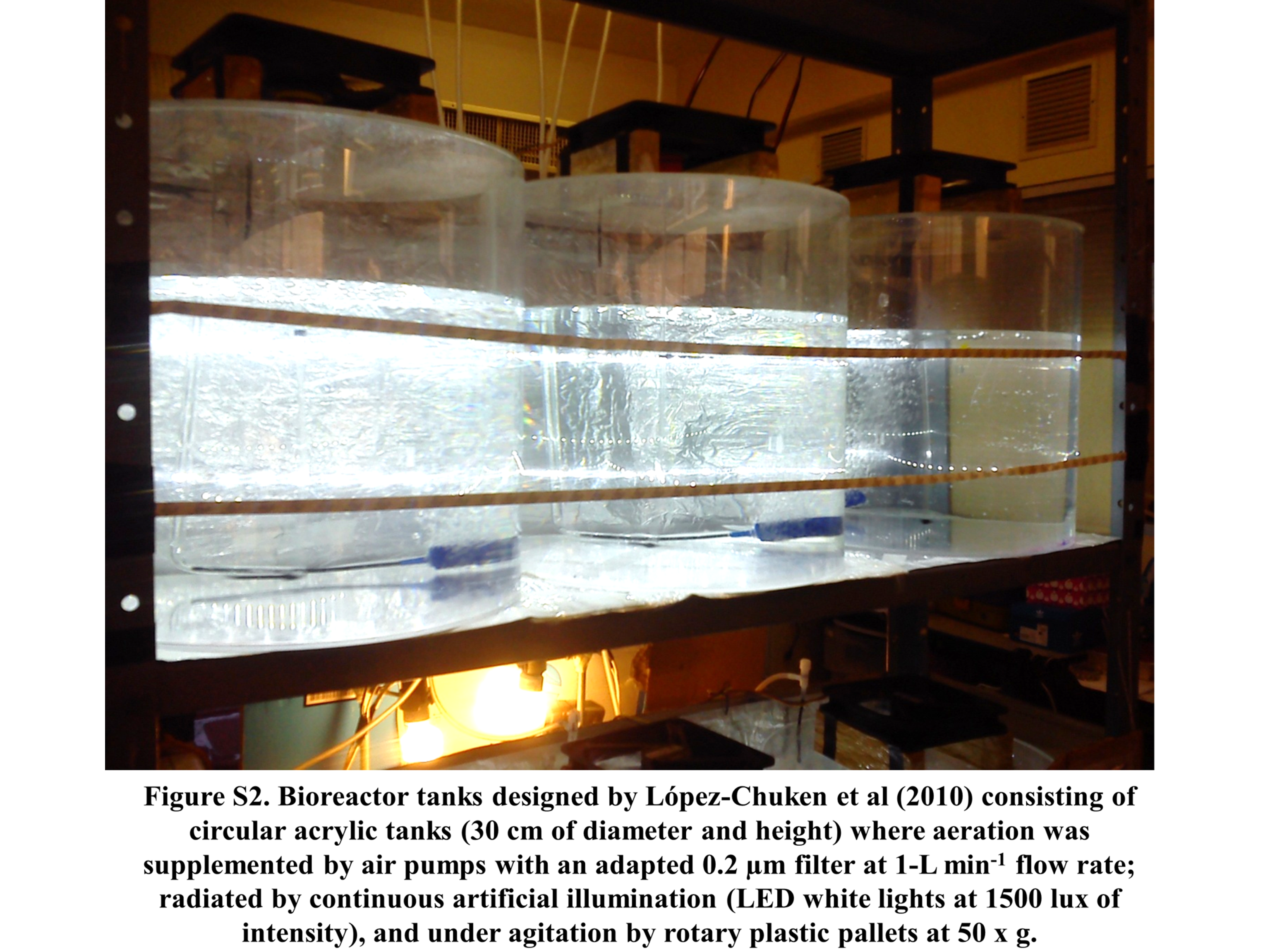

Supplement: Figure S2 — Bioreactor tanks designed by López-Chuken, Young & Guzman-Mar (2010) consisting of circular acrylic tanks (30 cm of diameter and height) where aeration was supplemented by air pumps with an adapted 0.2 µm filter at 1-L min-1 flow rate; radiated by continuous artificial illumination (LED white lights at 1,500 lux of intensity), and under agitation by rotary plastic pallets at 50× g. [file peerj-06-4358-s002.png]

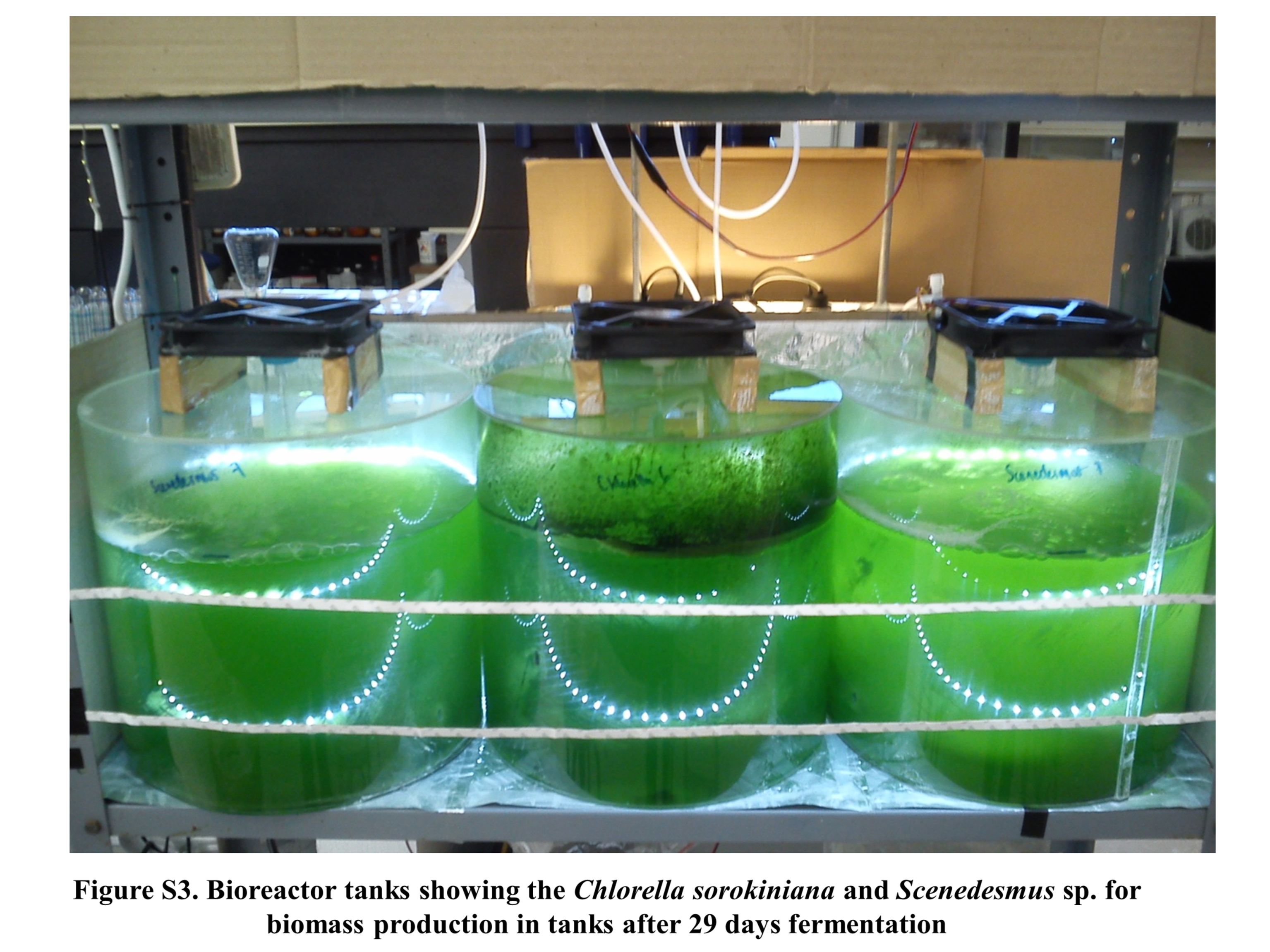

Supplement: Figure S3 — Bioreactor tanks showing the Chlorella sorokinian a and Scenedesmus sp. for biomass production in tanks after 29 days fermentation. [file peerj-06-4358-s003.png]
